# Determinants of Left Atrial Volume in Patients with Atrial Fibrillation

**DOI:** 10.1371/journal.pone.0164145

**Published:** 2016-10-04

**Authors:** Matthias Bossard, Rahel Kreuzmann, Thomas Hochgruber, Philipp Krisai, Andreas J. Zimmermann, Stefanie Aeschbacher, Katrin Pumpol, Arnheid Kessel-Schaefer, Frank-Peter Stephan, Nadja Handschin, Christian Sticherling, Stefan Osswald, Beat A. Kaufmann, Guillaume Paré, Michael Kühne, David Conen

**Affiliations:** 1 Division of Cardiology, Hamilton General Hospital, Hamilton Health Sciences, McMaster University, 237 Barton Street East, Hamilton, ON, L8L 2X2, Canada; 2 Population Health Research Institute, David Braley Cardiac, Vascular and Stroke Research Institute, McMaster University, 237 Barton Street East, Hamilton, ON, L8L 2X2, Canada; 3 Cardiology Division, Department of Medicine, University Hospital Basel, Petersgraben 4, 4031, Basel, Switzerland; 4 Cardiovascular Research Institute Basel, University Hospital Basel, Spitalstrasse 2, 4031, Basel, Switzerland; 5 Division of Internal Medicine, Department of Medicine, University Hospital Basel, Petersgraben 4, 4031, Basel, Switzerland; 6 Department of Pathology and Molecular Medicine, McMaster University, Michael G. DeGroote School of Medicine, 1280 Main Street West, Hamilton ON, L8S 4K1, Canada; Kurume University School of Medicine, JAPAN

## Abstract

**Introduction:**

Left atrial (LA) enlargement is an important risk factor for incident stroke and a key determinant for the success of rhythm control strategies in patients with atrial fibrillation (AF). However, factors associated with LA volume in AF patients remain poorly understood.

**Methods:**

Patients with paroxysmal or persistent AF were enrolled in this study. Real time 3-D echocardiography was performed in all participants and analyzed offline in a standardized manner. We performed stepwise backward linear regression analyses using a broad set of clinical parameters to determine independent correlates for 3-D LA volume.

**Results:**

We included 210 patients (70.9% male, mean age 61±11years). Paroxysmal and persistent AF were present in 95 (45%) and 115 (55%) patients, respectively. Overall, 115 (55%) had hypertension, 11 (5%) had diabetes, and 18 (9%) had ischemic heart disease. Mean indexed LA volume was 36±12ml/m^2^. In multivariable models, significant associations were found for female sex (β coefficient -10.51 (95% confidence interval (CI) -17.85;-3.16), p = 0.0053), undergoing cardioversion (β 11.95 (CI 5.15; 18.74), p = 0.0006), diabetes (β 14.23 (CI 2.36; 26.10), p = 0.019), body surface area (BSA) (β 34.21 (CI 19.30; 49.12), p<0.0001), glomerular filtration rate (β -0.21 (CI -0.36; -0.06), p = 0.0064) and plasma levels of NT-pro brain natriuretic peptide (NT-proBNP) (β 6.79 (CI 4.05; 9.52), p<0.0001), but not age (p = 0.59) or hypertension (p = 0.42). Our final model explained 52% of the LA volume variability.

**Conclusions:**

In patients with AF, the most important correlates with LA volume are sex, BSA, diabetes, renal function and NT-proBNP, but not age or hypertension. These results may help to refine rhythm control strategies in AF patients.

## Introduction

Atrial fibrillation (AF) is the most common sustained cardiac arrhythmia in the general population, and its prevalence is expected to increase in the coming decades.[1,2] AF has an important impact on general health in affected patients, because of its close relationship with heart failure, stroke, cognitive dysfunction and death.[1,2]

The pathophysiology of AF is complex and incompletely understood, but atrial remodelling and fibrosis seem to play a key role.[1,3,4] The left atrium (LA) has a reservoir and conduit function, and is an important regulator of left ventricular filling.[3] It also reflects an important electrophysiological substrate and has neurohumoral properties by releasing natriuretic peptides.[3,5] Those factors are closely related to left ventricular systolic and diastolic function.[3,6] LA enlargement itself is an important risk factor for incident AF[6–10], and is related to an increased stroke risk.[6–12] LA size is also a key determinant for the success of rhythm control strategies in patients with AF.[3,4] In the general population without established cardiac diseases, various factors influencing the LA volume have been described, including age, body size, body mass index and elevated blood pressure.[3,6,9,12–14]

In contrast, little evidence is available on determinants of LA volume among patients with established AF. This is an important issue, given the increasing use of pharmacological treatments and interventional procedures to maintain sinus rhythm in AF patients, and the fact that these strategies have limited long-term success rates and significant risks.[15,16] Thus, an enhanced understanding of factors associated with LA enlargement and atrial remodeling in this patient population may help to refine these strategies and improve success rates. Consequently, we assessed independent determinants of LA volume in patients with AF undergoing interventions aiming to achieve sinus rhythm.

## Methods

### Study population

We analyzed consecutive patients with either paroxysmal or persistent AF, who were scheduled to undergo catheter ablation (CA) (n = 140) or electrical cardioversion (ECV) (n = 80) at a tertiary academic center in Switzerland between January 2010 and March 2012. Paroxysmal AF was defined as AF consisting of episodes, which spontaneously converted within 7 days, and persistent AF as AF with at least one episode lasting >7 days.[1] Patients needed to be older than 18 years, provide informed consent and AF had to be confirmed by Holter-ECG. We excluded patients with severe valvular disease qualifying for interventional or surgical treatment, unstable and acute heart failure, limiting active or chronic major diseases, and a history of open-heart surgery within 3 months before enrollment. The local ethics committee Ethikkommission Nordwest- und Zentralschweiz (EKNZ) approved the study protocols. Each patient provided written informed consent. The study has been conducted according to the principles expressed in the Declaration of Helsinki. Due to consent form restrictions, data cannot be made publicly available and all analyses must be approved by the principal investigator.

### Study procedures

Electrocardiographic evidence of AF was obtained from every participant. All patients had to have appropriate oral anticoagulation before, during and after CA or ECV. ECV and CA procedures were performed with patients under conscious sedation in conformity with local recommendations and clinical standards. An experienced echocardiographer acquired a real-time three-dimensional transthoracic echocardiogram (RT3DE) in all patients within 24 hours prior to the corresponding intervention. Of the 220 patients enrolled, 10 were excluded due to missing data, leaving 210 patients for the current analyses.

### Assessment of clinical and blood parameters

Information about personal and medical factors was obtained using standardized questionnaires. Prior to the intervention, fasting venous blood samples were collected in EDTA containers, immediately centrifuged and stored at -80°C. Serum creatinine, cystatin C, high-sensitivity C-reactive protein, and interleukin 6 (IL-6) were measured on a Beckman Coulter Unicel DxC600 Synchron Clinical System (Beckman Coulter, California). High-sensitivity troponin T (cTnT) and N-terminal proB-type natriuretic peptide (NT-proBNP) were assessed on the Elecsys 2010 immunoassay analyzer (F. Hoffmann–La Roche, Switzerland). The body surface was estimated by using Du Bois’s equation (Body surface area (m^2^) = [Weight (kg)^0.425^ x height (m)^0.725^] x 0.007184). The creatinine and cystatine C based chronic kidney disease epidemiology collaboration (CKD-EPI) formula was applied to estimate the glomerular filtration rate (eGFR).[17]

### Echocardiographic image acquisition and quantification

Echocardiograms were performed by three dedicated cardiologists with an iE33 (Philips Medical Systems, Andover, MA) equipped with a X3-1 and X5-1 transducer. Two-dimensional cine-loops of the parasternal long and short axis, as well as of the apical two-, three- and four-chamber views were obtained in each patient. For RT3DE imaging, full volume loops were collected during breath-hold with gated acquisition, and with sector size and depth optimized to obtain the maximum possible frame rate. Gain settings were adjusted to enable specific adjustments during post-processing, if necessary. For LA volume assessment the image view was adapted if needed to achieve optimal delineation of LA structures, and the trigger delay was set to 300ms for coverage of the entire ventricular diastole. At least two RT3DE datasets were collected per patient and transferred to a workstation for offline analysis by a trained cardiologist using dedicated three-dimensional quantification software (4D-LA-analysis, TomTec-Imaging Systems, Unterschleissheim Munich, Germany).[18–20]

For RT3DE analysis of the LA, initial LA contours at end-diastole and end-systole were manually defined for the apical four-chamber, two-chamber, and long-axis views excluding the LA appendage and pulmonary veins.[18–20] The software then constructed a three-dimensional polyhedral model of the LA by an automated border detection approach and calculated the maximal (VMax [ml]) and minimal (VMin [ml]) LA volumes.[18–20]

All analyses were performed in a blinded fashion by two physicians (A.Z. and M.B.). Interobserver agreement was determined by using repeated VMax measurements from 10 randomly selected subjects 12 months after the first analysis, with a mean difference for VMax of 9±7ml.

### Statistical analysis

All statistical analyses were done using SAS statistical software (version 9.4; Cary, NC). A p-value of <0.05 was pre-specified to indicate statistical significance. Baseline characteristics were stratified according to the underlying AF type. The distribution patterns for continuous variables were evaluated using kurtosis, skewness and visual inspection of the histogram. Continuous data were expressed as mean ± SD or median (interquartile range), categorical data as frequency (percentage). Normally distributed variables were compared using unpaired Student t-tests, otherwise Wilcoxon’s rank sum tests were used. Categorical variables were compared by Chi-square or Fisher’s exact tests, as appropriate. All analyses were performed using maximal absolute LA volume (Vmax) as a continuous variable (in mL). Categorical variables were entered in the multivariable models using binary indicator variables.

First, all continuous covariates were grouped into quartiles to assess the linearity of their relationship with Vmax in univariable linear regression models. Variables with non-linear associations were entered as categorical variables in the subsequent regression models. Second, stepwise backward linear regression analyses were performed to identify covariates independently associated with LA volume (Vmax), using a p-value <0.05 as a threshold to stay in the model. A list of 17 variables was used for this analysis, all of them being significantly associated with LA volume in the univariable models: Sex, age, cohort, AF type (paroxysmal versus persistent), arterial hypertension, diabetes, sleep apnea syndrome, history of heart failure, moderate or severe mitral regurgitation, body surface area, heart rate, IL-6, NTproBNP, cTnT ≥15ng/L, eGFR, left ventricular mass and left ventricular ejection fraction. Third, subgroup analyses according to AF type were performed to evaluate consistency of the relationships between a set of independent covariates and LA volume. Formal differences across subgroups were assessed using multiplicative interaction terms in the non-stratified models.

## Results

Mean age of the 210 patients enrolled was 61 years and 149 participants (70.9%) were male. Baseline characteristics according to AF type are presented in *Table 1*. Overall, 95 (45.2%) and 115 (54.8%) participants had paroxysmal and persistent AF, respectively. The median time from AF diagnosis was 1 year (interquartile range 1; 5). The mean absolute and indexed LA volumes were 72.2±26.8ml and 36.5±12.5ml/m^2^, respectively. Patients with persistent AF were older and had more comorbidities, including a higher prevalence of hypertension, diabetes, ischemic heart disease, mitral regurgitation and history of valve replacement. Patients with paroxysmal AF had a lower heart rate, a lower diastolic blood pressure, lower levels of IL-6 and NT-proBNP and a higher eGFR. Left atrial enlargement defined as >34ml/m^2^ was found in 105 (50%) patients.

**Table 1:** Baseline characteristics according to underlying atrial fibrillation type.

*(n = 210)*	*Paroxysmal AF (n = 95)*	*Persistent AF*^†^*(n = 115)*	P-value^*^
Indexed left atrial volume (ml/m^2^)	28 (24; 33)	42 (33; 50)	*<0*.*0001*
Age (years)	60 (54; 66)	65 (54; 71)	*0*.*058*
Males (%)	63 (66.3)	86 (74.8)	*0*.*18*
Heart rate (bpm)	60 (52; 68)	80 (71; 92)	*<0*.*0001*
Systolic blood pressure (mmHg)	131 (120; 142)	134 (119; 149)	*0*.*48*
Diastolic blood pressure (mmHg)	80 (73; 87)	87 (73; 100)	*0*.*007*
Body mass index (kg/m^2^)	25.1 (23.6; 28.1)	27.4 (25.0; 30.5)	*0*.*0002*
Body surface area (m^2^) ^‡^	1.96 (1.76; 2.08)	1.99 (1.84; 2.13)	*0*.*027*
Arterial hypertension (%)	44 (46.3)	71 (61.7)	*0*.*025*
Diabetes mellitus (%)	0 (0)	11 (9.6)	*0*.*002*
Ischemic heart disease (%)	3 (3.2)	15 (13.0)	*0*.*011*
History of heart failure (y/n)	4 (4.2)	16 (13.9)	*0*.*017*
Present moderate or severe MR (%)	2 (2.1)	16 (13.9)	*0*.*002*
History of valve replacement (%) ^¶^	0 (0)	5 (4.4)	*0*.*039*
History of stroke (%) ^§^	10 (10.5)	11 (9.6)	*0*.*82*
Sleep apnea syndrome (%)	2 (2.1)	13 (11.3)	*0*.*010*
*Echocardiographic parameters*:			
LVEF (%)	53 (48; 59)	45 (35; 52)	*<0*.*0001*
Indexed LV mass (g/m^2^)	89 (73; 107)	101 (83; 122)	*0*.*0011*
*Laboratory parameters*:			
eGFR (ml/min1.73m^2^) ^*°*^	92 (83; 104)	88 (73; 98)	*0*.*003*
Interleukin 6 (pg/mL)	1.65 (1.18; 2.46)	2.24 (1.65; 3.38)	*<0*.*0001*
NT-proBNP (pg/mL)	16.9 (9.5; 35.6)	114 (64; 181)	*<0*.*0001*
High-sensitivity TnT (ng/L)	4.07 (1.50; 7.01)	4.85 (1.50; 9.89)	*0*.*086*

Data are median (interquartile range) or number (percentage). LVEF = Left ventricular ejection fraction. LV = Left ventricular. eGFR = estimated glomerular filtration rate; bpm = Beat per minute; MR = Mitral regurgitation; NT-proBNP = N-terminal-pro B-type natriuretic peptide; TnT = Troponin T.

^*^ P values were based on student’s t-tests, Mann-Whitney U- tests or Chi-square tests, as appropriate.

^†^ 37 patients with persistent atrial fibrillation were scheduled for pulmonary vein isolation.

^‡^ The body surface (m^2^) was estimated by applying Du Bois’ method.

^¶^ Includes any surgical valve replacement in the past.

^§^ Emphasizes any minor or major stroke in the past.

^*°*^ The estimated glomerular filtration rate was calculated by using the creatinine and cystatine based CKD-EPI formula.

### Independent determinants of left atrial volume

**Fig 1:**
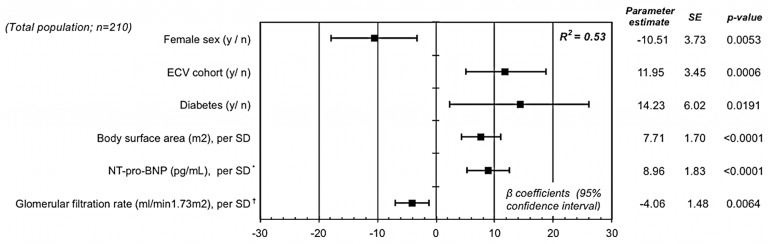
Independent determinants of left atrial volume in AF patients. SE = Standard error; SD = Standard deviation; y/n = yes / no; ECV = Electrical cardioversion; NT-proBNP = N-terminal-pro B-type natriuretic peptide; eGFR = Estimated glomerular filtration rate. R2 is reported for the final multivariable model. The β (95% confidence intervals) represents the increase or decrease in left atrial volume (mL) per unit change of the specific covariate. The multivariable model also included age, atrial fibrillation type (paroxysmal vs. persistent), resting heart rate, left ventricular ejection fraction, left ventricular mass, high-sensitivity troponin T ≥15ng/mL, interleukin-6, history of heart failure, arterial hypertension, moderate or severe mitral regurgitation and sleep apnea syndrome. All above presented variables selected by the stepwise backward regression model were significant at the ≤0.05 level. * log-transformed variables. ^†^ Estimated by the CKD-EPI formula including creatinine and cystatin C.

Variables significantly associated with LA volume in univariable regression analyses are shown in the *S1 Table.* Using backward multivariable regression analyses, we found six independent factors associated with absolute LA volume, as illustrated in *Fig 1*. The strongest correlates of LA volume were body surface area (β-regression coefficient 7.71 per SD (95% confidence interval (CI) 4.35; 11.06)) and NT-proBNP (β 8.96 per SD (95% CI 5.35; 12.58)), p for all <0.0001. Additionally, we found significant associations of LA volume with the female gender (β -10.51 (95% CI -17.85; -3.16), p = 0.0053) the ECV cohort (β 11.95 (95% CI 5.15; 18.74), p = 0.0006), diabetes (β 14.23 (95% CI 2.35; 26.10), p = 0.0191) and eGFR (β -4.06 per SD (95% CI -6.97; -1.15), p = 0.0064). The R^2^ of our final multivariate model was 0.52, suggesting that our model captured 52% of the overall LA volume variability in our sample. To assess the cohort effect among our models, we performed an additional multivariable analysis excluding this factor. This analysis revealed that persistent AF seems to be also an independent determinant of LA volume (p = 0.014), as shown in *S1 Fig*. Subgroup analyses stratified by AF type showed consistent associations of these risk factors with LA volume among those with paroxysmal and persistent AF (all p values for interaction = NS), as shown in *Table 2*.

**Table 2:** Subgroup analyses for independent determinants of left atrial volume stratified by atrial fibrillation type.

*(n = 210)* *	Left atrial volume (mL)	
	β (95%CI)	*P for interaction*
Sex (y/n)		
Paroxysmal AF	-7.12 (-16.12; -1.88)	0.66
Persistent AF	-13.38 (-25.12; -1.65)	
Body surface area (m^2^), per SD		
Paroxysmal AF	9.17 (5.08; 13.26)	0.95
Persistent AF	8.36 (3.18; 13.55)	
NT-proBNP, log-unit (pg/mL), per SD ^†^		
Paroxysmal AF	7.92 (3.58; 12.26)	0.18
Persistent AF	12.26 (5.58; 18.95)	
eGFR (ml/min 1.73m^2^), per SD		
Paroxysmal AF	-2.83 (-7.03; 1.36)	0.24
Persistent AF	-4.87 (-9.17; 0.57)	

AF = Atrial fibrillation; y/n = yes / no; SD = Standard deviation; NT-proBNP = N-terminal-pro B-type natriuretic peptide; eGRF = Estimated glomerular filtration rate. The β (95% confidence intervals) represents the increase or decrease in left atrial volume (mL) per unit change of the specific covariate. The multivariable models included sex, body surface area, estimated glomerular filtration rate and N-terminal-pro B-type natriuretic peptide.

* Paroxysmal and persistent AF were found in 95 and 115 patients, respectively.

^†^ log-transformed variables

## Discussion

In patients with AF, assessment of LA dimensions is fundamental for follow-up and outcome prediction, including determination of arrhythmic freedom, quality of life and thromboembolic risk.[10] In order to define independent predictors of LA volume that may help to improve risk stratification in this context, we assessed LA volume with RT3DE in a large sample of patients with symptomatic AF. In a model that explained more than 50% of the LA volume variability, we found that LA volume was independently associated with sex, need for an ECV, presence of diabetes, BMI, NT-proBNP and renal function. To the best of our knowledge, this is one of the first studies to comprehensively assess determinants of LA volume in patients with manifest AF.

Our results show a substantially larger LA volume in male AF patients, confirming and expanding earlier data from healthy populations.[21] Interestingly, the incidence of AF is lower in women than in men at every age group, which may be explained by the fact that a small LA may protect from developing AF.[12] Further studies are needed to evaluate whether this finding is due to smaller body size in women or to true sex specific differences.

Body dimensions and diabetes are important metabolic risk factors for AF.[22–25] The body dimensions, height and weight, have been shown to be a major determinant of LA size in the general population.[6,13,14] Our study confirms the importance of obesity as a key risk factor for LA enlargement in patients with established AF. The interrelationships of body dimensions (e.g. body mass index), LA dimensions and incident AF have been shown previously.[12,13,22,24,26] In addition, a recent study showed the relevance of weight reduction for the reduction of both AF related symptoms and LA dimensions.[26] Thus, our study and the available evidence underscore the importance of weight loss in patients with symptomatic AF. Interestingly, diabetes seems to have a detrimental effect on LA volume that is independent of obesity.[27,28] Diabetes has been associated with inflammatory processes, cardiac fibrosis and diastolic dysfunction, which in turn may promote LA remodeling and atrial arrhythmogenicity independent of body fat.[4,28]

Natriuretic peptides are strong and independent predictors of incident AF and subsequent outcomes.[5,29–31] Two smaller studies have suggested correlations between NT-proBNP and LA volume among AF patients undergoing CA.[31,32] Our study expands those findings to a broader AF population, and implicates that NT-proBNP may not only be a marker for heart failure, but also a useful surrogate for LA volume estimation in patients with AF. In addition, our results suggest that renal function is independently associated with LA volume in AF patients, which to our knowledge has not been shown yet, and which may be related to fluid retention in individuals with worse renal function.

Patients undergoing ECV had a larger LA volume compared to CA patients, independent of AF duration and the patient’s age. This finding is potentially attributable to a persistently elevated heart rate, a depressed left ventricular function and higher prevalence of underlying structural heart disease; underscoring the importance of aggressively managing risk factors and co-morbidities in AF patients.

One of the key findings of our analyses is that age and hypertension, which are important risk factors for LA enlargement or incident AF in the general population, may not independently influence LA volume and remodelling in AF patients.[1,11,12,33] These contrasting findings might emphasize the complex pathophysiology of LA enlargement. While in individuals without AF, age and hypertension may be important drivers of LA remodelling, other factors seem to be more important in individuals with AF whose LA’s presumably already have undergone a significant remodelling process. These factors are highlighted in the current study and include BMI, metabolic profile and renal function, all of which are associated with volume overload, adverse hemodynamic changes, autonomic dysfunction, higher levels of pro-inflammatory cytokines and a stimulated renin-aldosterone-angiotensin-system.[4,12,22,24,26–28,34–36] In this context, our data do certainly not mean that blood pressure control should be neglected in AF patients.

There are limitations of this study that deserve mentioning. First, certain patients were in AF while RT3DE was acquired; however, this technology is not thoroughly validated for assessment of cardiac function and structure during AF.[18–20,37] But, given the superior reproducibility of RT3DE over time in contrast to conventional 2D methods in sinus rhythm, it is plausible that the same is true in patients with AF.[18–20,37,38] Second, our results do apply to patients with AF, and generalizability to those without AF remains unclear. Third, aging plays an important role in the context of AF development and the mean age of our patients was rather low. It is therefore unknown, if our results are also applicable to older patients with AF. Finally, the cross-sectional study design does not allow drawing any causal inferences.

## Conclusions

Using RT3DE, we found that in patients with AF, the most important correlates with LA volume are sex, body surface area, diabetes, renal function and NT-proBNP. Age and hypertension might play a less important role in this context. These results may help to understand the pathophysiology of LA enlargement and define targeted intervention strategies in AF patients.

## Supporting Information

S1 Fig Assessment of independent determinants of left atrial volume in AF patients–Analysis excluding the cohort factor. SE = Standard error; SD = Standard deviation; y/n = yes / no; AF = Atrial fibrillation; NT-proBNP = N-terminal-pro B-type natriuretic peptide; eGFR = Estimated glomerular filtration rate. R2 is reported for the final multivariable model. The β (95% confidence intervals) represents the increase or decrease in left atrial volume (mL) per unit change of the specific covariate. The multivariable model also included age, resting heart rate, left ventricular ejection fraction, left ventricular mass, high-sensitivity troponin T ≥15ng/mL, interleukin-6, history of heart failure, arterial hypertension, moderate or severe mitral regurgitation and sleep apnea syndrome. All above presented variables selected by the stepwise backward regression model were significant at the ≤0.05 level. * log-transformed variables. ^†^ Estimated by the CKD-EPI formula including creatinine and cystatin C.
(TIF)

S1 Table Associations between left atrial volume and various variables in univariate analyses. AF = Atrial fibrillation; y/n = yes / no; PVI = Pulmonary vein isolation; bpm = beat per minute; ECV = Electrical cardioversion; MR = Mitral regurgitation; NT-proBNP = N-terminal-pro B-type natriuretic peptide; eGRF = Estimated glomerular filtration rate. The β (95% confidence intervals) represents the increase or decrease in left atrial volume (mL) per unit change of the specific covariate. * Paroxysmal and persistent AF were found in 95 and 115 patients, respectively. ^†^ Emphasizes any minor or major stroke in the past. ^‡^ The estimated glomerular filtration rate was calculated by using the creatinine and cystatine based CKD-EPI formula. ^¶^ log-transformed variables.
(DOCX)
