# Supplementary figures and images for: Determinants of Left Atrial Volume in Patients with Atrial Fibrillation
Source: PLoS One. 2016 Oct 4;11(10):e0164145. doi: 10.1371/journal.pone.0164145 (PMC5049755; doi:10.1371/journal.pone.0164145)

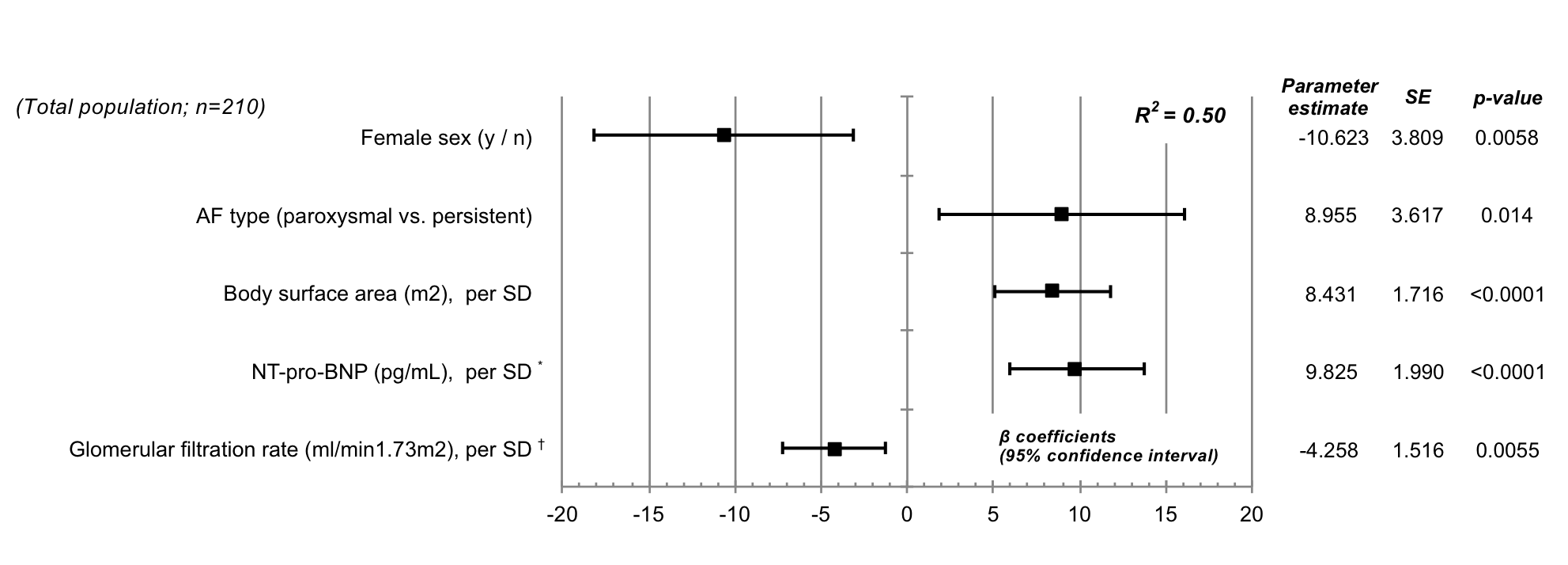

Supplement: S1 Fig — SE = Standard error; SD = Standard deviation; y/n = yes / no; AF = Atrial fibrillation; NT-proBNP = N-terminal-pro B-type natriuretic peptide; eGFR = Estimated glomerular filtration rate. R2 is reported for the final multivariable model. The β (95% confidence intervals) represents the increase or decrease in left atrial volume (mL) per unit change of the specific covariate. The multivariable model also included age, resting heart rate, left ventricular ejection fraction, left ventricular mass, high-sensitivity troponin T ≥15ng/mL, interleukin-6, history of heart failure, arterial hypertension, moderate or severe mitral regurgitation and sleep apnea syndrome. All above presented variables selected by the stepwise backward regression model were significant at the ≤0.05 level. * log-transformed variables. † Estimated by the CKD-EPI formula including creatinine and cystatin C. (TIF) [file pone.0164145.s001.tif]
