# Supplementary material for: Determinants of Left Atrial Volume in Patients with Atrial Fibrillation
Source: PLoS One. 2016 Oct 4;11(10):e0164145. doi: 10.1371/journal.pone.0164145 (PMC5049755; doi:10.1371/journal.pone.0164145)
Supplement: S1 Table — AF = Atrial fibrillation; y/n = yes / no; PVI = Pulmonary vein isolation; bpm = beat per minute; ECV = Electrical cardioversion; MR = Mitral regurgitation; NT-proBNP = N-terminal-pro B-type natriuretic peptide; eGRF = Estimated glomerular filtration rate. The β (95% confidence intervals) represents the increase or decrease in left atrial volume (mL) per unit change of the specific covariate. * Paroxysmal and persistent AF were found in 95 and 115 patients, respectively. † Emphasizes any minor or major stroke in the past. ‡ The estimated glomerular filtration rate was calculated by using the creatinine and cystatine based CKD-EPI formula. ¶ log-transformed variables. (DOCX) [file pone.0164145.s002.docx]

**S1 Table. Associations between left atrial volume and various variables in univariate analyses**

| *(n=210)* ^*^ | **Left atrial volume (mL)** | |
| --- | --- | --- |
|  | **β (95%CI)** | ***P-value****^*^* |
| Sex (y/n) | -18.28 (-25.93; -10.63) | <0.0001 |
| Age (years) | 0.55 (0.22; 0.89) | 0.0013 |
| AF cohort (PVI vs. ECV) | 27.74 (21.18; 34.30) | <0.0001 |
| AF type (paroxysmal vs. persistent) ^*^ | 26.62 (20.25; 32.99) | <0.0001 |
| Time since AF diagnosis (years) | -2.16 (-4.42; 0.08) | 0.059 |
| Current smoking (y/n) | -10.26 (-22.62; 2.09) | 0.10 |
| Heart rate (bpm) | 0.36 (0.18; 0.54) | <0.0001 |
| Systolic blood pressure (mmHg) | 0.14 (-0.04; 0.32) | 0.14 |
| Diastolic blood pressure (mmHg) | 0.39 (0.14; 0.63) | 0.0019 |
| Body surface area (m^2^) | 49.44 (34.66; 64.22) | <0.0001 |
| Arterial hypertension (%) | 11.00 (3.82; 18.19) | 0.0028 |
| Diabetes mellitus (%) | 27.21 (11.24; 43.17) | 0.0009 |
| Ischemic heart disease (y/n) | 11.69 (-1.25; 24.64) | 0.076 |
| History of heart failure (y/n) | 18.57 (6.39; 30.75) | 0.0030 |
| History of stroke (y/n) ^†^ | 8.87 (-3.23; 20.99) | 0.15 |
| Present moderate or severe MR (y/n) | 17.65 (4.83; 30.47) | 0.0072 |
| History of valve replacement (y/n)^‡^ | -6.79 (-30.73; 17.14) | 0.57 |
| Sleep apnea syndrome (y/n) | 16.83 (2.84; 30.82) | 0.018 |
| *Echocardiographic parameters:* |  |  |
| LVEF (%) | -0.77 (-1.05; -0.48) | <0.0001 |
| LV mass (g) | 0.09 (0.04; 0.14) | 0.0002 |
| Relative wall thickness | -10.59 (-39.60; 18.41) | 0.47 |
| *Laboratory parameters:* |  |  |
| eGFR (ml/min1.73m^2^) ^‡^ | -0.45 (-0.63; -0.27) | <0.0001 |
| Interleukin 6 (pg/mL) ^¶^ | 13.55 (8.82; 18.28) | <0.0001 |
| NT-proBNP (pg/mL) ^¶^ | 9.93 (7.52; 12.35) | <0.0001 |
| High-sensitivity TnT ≥15ng/mL (y/n) | 26.69 (15.33; 38.04) | <0.0001 |
| C-reactive protein >2mg/L (y/n) | 0.41 (-6.96; 7.79) | 0.91 |
|  | |  |

AF = Atrial fibrillation; y/n = yes / no; PVI = Pulmonary vein isolation; bpm = beat per minute; ECV = Electrical cardioversion; MR = Mitral regurgitation; NT-proBNP = N-terminal-pro B-type natriuretic peptide; eGRF = Estimated glomerular filtration rate. The β (95% confidence intervals) represents the increase or decrease in left atrial volume (mL) per unit change of the specific covariate.

^*^ Paroxysmal and persistent AF were found in 95 and 115 patients, respectively.

^†^ Emphasizes any minor or major stroke in the past.

^‡^ The estimated glomerular filtration rate was calculated by using the creatinine and cystatine based CKD-EPI formula.

^¶^ log-transformed variables
